# Roles of the phagocytosis checkpoint in radiotherapy

**DOI:** 10.1038/s41419-025-07921-5

**Published:** 2025-08-20

**Authors:** Yulan Kui, Fan Tong, Ruiguang Zhang, Jian Wang, Xiaorong Dong

**Affiliations:** 1 Cancer Center, Union Hospital, Tongji Medical College, Huazhong University of Science and Technology, Wuhan, Hubei 430022 China; 2 Hubei Key Laboratory of Precision Radiation Oncology, Wuhan, Hubei 430022 China; 3 Institute of Radiation Oncology, Union Hospital, Tongji Medical College, Huazhong University of Science and Technology, Wuhan, Hubei 430022 China

**Keywords:** Immunoediting, Radiotherapy

## Abstract

Radiotherapy is widely used in cancer treatment in both curative and palliative care due to its good safety profile and broad clinical availability. It not only directly destroys tumor cells by damaging their DNA but also plays a critical immunomodulatory role, making it a potential combination partner for immunotherapy. Radiotherapy-induced immune effects are complex. They could enhance antitumor immunity by releasing tumor antigens but also promote tumor immune evasion by adaptively regulating immunosuppressive molecules, such as phagocytosis checkpoints. However, the effects of radiotherapy on phagocytosis checkpoints are not fully elaborated compared to T cell-associated immune checkpoints. Phagocytosis checkpoints are regulated by a series of receptor-ligand binding molecules, respectively on the tumor cells and phagocytes, which mediate pro-phagocytosis or anti-phagocytosis signals, modulate tumor antigen presentation, and further determine the infiltration of tumor-specific cytotoxic T cells in the tumor microenvironment. Radiotherapy regulates the different phagocytosis checkpoints on the tumor cells and phagocytes to modulate phagocytic clearance and reshape the irradiated tumor microenvironment. Therefore, radiotherapy in combination with phagocytosis checkpoints-associated immunotherapy can be a promising antitumor approach by considering the type, dose, and sequence of this combinatory regimen as well as the biomarkers for patient selection. This review attempts to summarize the cross-effects of radiotherapy and phagocytosis checkpoints and their combination strategies to enhance the efficiency of radiotherapy and improve the survival of cancer patients. Opportunities built on the roles of the phagocytosis checkpoint in radiotherapy are duly warranted.

## Facts

- As a bridge between innate and adaptive immunity, the phagocytosis checkpoints play important roles both in antitumor immunity and in cancer immune evasion.
- Radiotherapy can impact phagocytosis checkpoints through diverse molecular mechanisms.
- Radiotherapy-induced changes in phagocytosis checkpoints influence the anti-tumor effects of radiotherapy.
- Radiotherapy combined with phagocytosis checkpoints-associated immunotherapy is a novel and promising treatment strategy.

## Open Questions

- What are the detailed mechanisms by which radiotherapy regulates phagocytosis checkpoints?
- What is the impact of radiotherapy on the tumor immune microenvironment after radiotherapy-induced changes in phagocytosis checkpoints?
- What is the effect of phagocytosis checkpoints-associated immunotherapy on the anti-tumor efficacy of radiotherapy?
- How to combine radiotherapy and phagocytosis checkpoint-associated immunotherapy to boost antitumor effect?

## Introduction

As a well-established therapeutic modality, radiotherapy plays an important role in the local treatment of various cancers, whether it is used alone or in conjunction with other treatments [1]. Radiotherapy directly leads to tumor tissue apoptosis by causing lethal DNA damage [2]. Radiotherapy also regulates complicated immune responses, including both innate and adaptive immune responses. For example, radiotherapy promotes innate immunity via MHC-I-dependent mechanisms [3], cell surface death receptor FAS [4], death receptor DR5 [4], damage-associated molecular patterns (DAMPs) [5], and calreticulin [6, 7]. Radiotherapy also enhances adaptive immunity through interferon γ (IFN-γ) [8], tumor necrosis factor α (TNF-α) [9], and activation of tumor-specific T cells [5, 8, 9]. Furthermore, radiotherapy has a systemic abscopal effect, leading to anti-tumor response for non-irradiated tumor tissues [10, 11]. However, radiotherapy can also induce immunosuppressive effects, such as systemic and intratumoral lymphopenia [12], transforming growth factor β(TGFβ) [13], immunosuppressive M2-phenotype macrophages [14], Treg cells [15], and myeloid-derived suppressor cells (MDSCs) [16], resulting in radioresistance.

Immunotherapy is a powerful systemic antitumor treatment by activating patients’ immune systems to kill tumor cells [17]. Especially, the immune checkpoint blockade represented by targeting programmed death 1 ligand 1 (PD-L1)-programmed cell death 1 (PD-1) [18] and cytotoxic T-lymphocyte-associated protein 4 (CTLA-4) [19] is broadly applied in different tumors by activating antitumor T cells. Recently, innate immunotherapy has also been gradually applied in diverse tumors, especially phagocytosis checkpoints-associated immunotherapy [20–22]. Phagocytosis checkpoints are emerging as key mechanisms with the ability to inhibit or promote phagocytosis [23]. By suppressing or promoting these phagocytosis checkpoints helps cancer cells to escape or succumb to immune surveillance. In particular, they regulate the cytoplasmic regions of phagocyte receptors, such as immune receptor tyrosine-based inhibitor motifs (ITIMs) or immune receptor tyrosine-based activating motifs (ITAMs), and modulate their downstream signals [21]. The cluster of differentiation 47(CD47)–signal regulatory protein α (SIRPα) axis is the first identified anti-phagocytosis signal and is widely studied in numerous preclinical studies [24]. Moreover, increasing clinical trials targeting the CD47/SIRPα axis have been either finished or are ongoing and have achieved certain antitumor curative effects [25, 26]. Consequently, phagocytosis checkpoints as a crucial immune component can become novel diagnostic or prognostic biomarkers and promising therapeutic targets for cancer patients [27].

As mentioned above, T cell checkpoint-related immunotherapy is currently an essential pillar of tumor immunotherapy by reversing T cell exhaustion [28]. Therefore, in the field of radiotherapy combined with immunotherapy, numerous studies mainly focus on radiotherapy and T cell-mediated adaptive immunotherapy [29, 30]. However, radiotherapy can reduce the production of peripheral blood T cells because of the intrinsic sensitivity of the bone marrow and T cells themselves, which leads to lymphopenia and reduction of T cell infiltration in the tumor microenvironment [31, 32], resulting eventually in T-cell checkpoint-associated immunotherapy failure. This makes the combination treatment of radiotherapy and T cell-associated immunotherapy limited and suggests the need to find novel immunotherapy methods combined with radiotherapy [33]. Fortunately, radiotherapy has less impairment on macrophages because of the higher tolerance of macrophages to radiotherapy compared to T cells. Radiotherapy could also promote the recruitment of macrophages in the tumor microenvironment [34–36]. Furthermore, an increasing number of phagocytic checkpoints are being discovered [21], allowing for more selective targets to rely on the macrophages that remain after radiotherapy. Also, phagocytosis checkpoints are associated with dual immune responses, including innate and adaptive immunity. Therefore, as an emerging immunotherapy with both innate and adaptive immune functions, phagocytosis checkpoints-associated immunotherapy is a promising treatment option combined with radiotherapy. Importantly, accumulating evidence suggests that radiotherapy could regulate the expression of phagocytosis checkpoints [37–39].

Therefore, considering the promising application of phagocytosis checkpoints-associated immunotherapy combined with radiotherapy, we provide an overview of the regulatory mechanisms of diverse phagocytosis checkpoints upon radiotherapy. Then, we analyze the clinical application of phagocytosis checkpoints-associated immunotherapy and radiotherapy combination strategies in tumor patients. Altogether, this review aims to provide a molecular insight into the exploitation of phagocytosis checkpoints-associated immunotherapy combined with radiotherapy.

## The phagocytosis checkpoints in radiotherapy

**Fig. 1:**
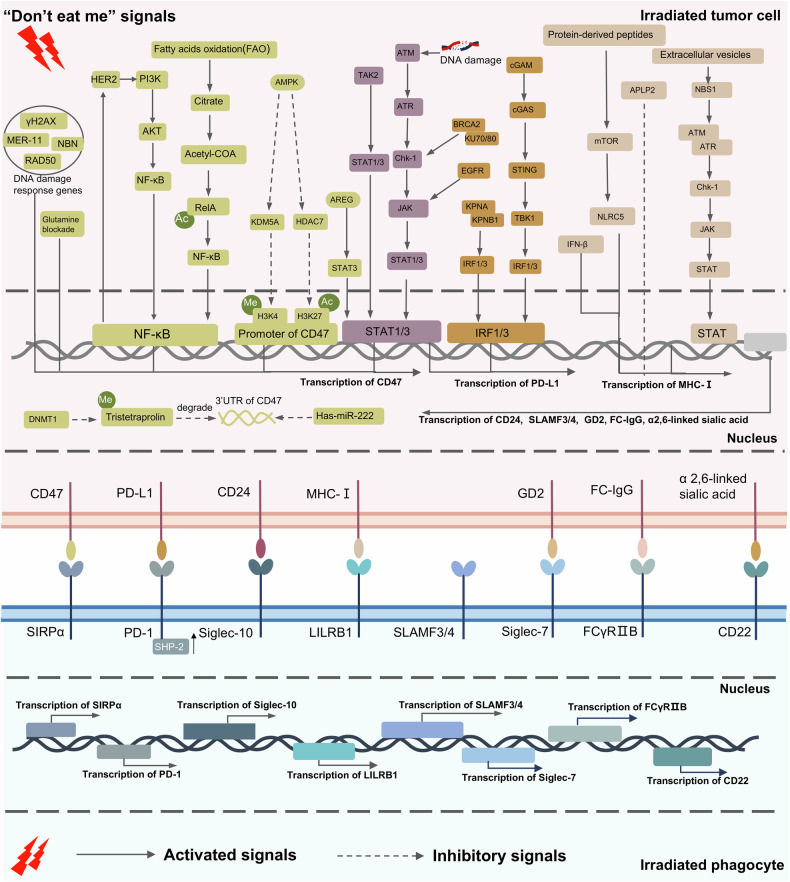
Regulation mechanisms of radiotherapy on “Don’t eat me” signals. In irradiated tumor cells, radiotherapy upregulates CD47 expression by DNA damage response-associated signals, HER2-PI3K/AKT-NF-κB axis, fatty acids oxidation-Acetyl-COA-NF-κB axis, AMPK-Histone modification signals, TAK2-STAT1/3 axis, AREG-STAT3 axis and ATM-ATR-JAK/STAT1/3 axis. At the post-transcriptional level, radiotherapy-suppressed Has-miR-222 and DNMT-TTP axis promotes CD47 expression. Also, the radiotherapy-induced PD-L1 upregulation is associated with DNA damage response-associated BRCA1/KU70/80-Chk-1-JAK-STAT1/3 axis, EGFR-JAK-STAT1/3 axis, cGAM-Cgas-STING-TBK1-IRF1/3 axis and KPNA/KPNB1-IRF1/3 axis. Furthermore, irradiated tumor cells-derived extracellular vesicles promote MHC-Iexpression through NBS1-ATM-ATR-JAK/STAT1/3 axis. Unfortunately, detailed regulation mechanisms of radiotherapy are unclear both on CD24, SLAMF3/4, GD2, FC-IgG and α2,6-linked sialic acid in irradiated tumor cells and on ligands of anti-phagocytosis in irradiated phagocytes according to current research. (Created with Microsoft Office PowerPoint).

**Fig. 2:**
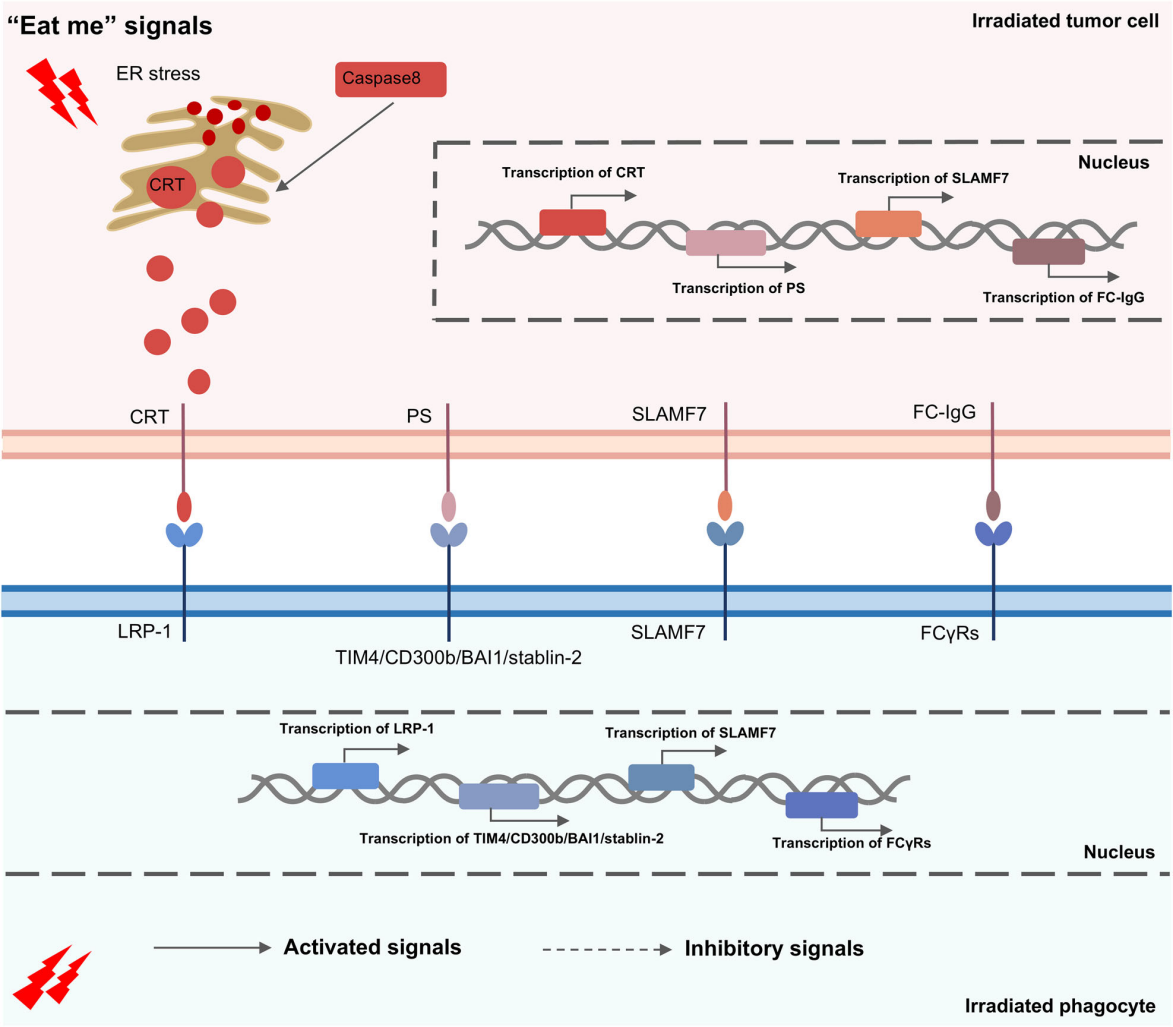
Regulation mechanisms of radiotherapy on “Eat me” signals. Radiotherapy promotes calreticulin translocation to the tumor cell surface by increasing endoplasmic reticulum (ER) stress in irradiated tumor cells. Radiotherapy-induced Caspase8 also promotes calreticulin translocation to irradiated tumor cell surface. However, regulation mechanisms of radiotherapy on other “Eat me” signals including Phosphatidylserine (PS)-TIM4/CD300b/BAI1/ Stabilin-2 axis, SLAMF7-SLAMF7 axis and Fc-FcγRs axis are still lacking. (Created with Microsoft Office PowerPoint).

Phagocytosis checkpoints have been gradually identified in recent years because of the fast development of high-throughput technologies allowing a deep understanding of cancer immunology and molecular bases. For example, the CD47/SIRPα axis was identified in the late 1990s as the first tumor phagocytosis checkpoint [20]. Next, others phagocytosis checkpoints including PD-L1-PD-1 axis (in 2017) [40], major histocompatibility complex class I (MHC-1)-leukocyte immunoglobulin-like receptor subfamily B member 1(LILRB1) axis (in 2018) [41], cluster of differentiation 24(CD24)-sialic acid binding Ig like lectin 10(Siglec-10) (in 2019) [42], cluster of differentiation 22 (CD22) (in 2019) [43], signaling lymphocytic activation molecule family 3/4 (SLAMF3/4) (in 2022) [44] and ganglioside 2 (GD2)-sialic acid binding Ig like lectin 7 (Siglec-7) (in 2022) [45] were followingly demonstrated in the tumor microenvironment. Radiotherapy can influence the phagocytosis checkpoints including the ligands on tumor cells, the receptors on the phagocytes and their downstream phagocytosis function signals (Table 1) (Figs. 1, 2).

**Table 1:** Mechanisms by which radiotherapy regulates phagocytosis checkpoints in tumors.

Signaling pathway	Regulatory mechanism	Tumor type	Clinical Translation	Ref.
CD47-SIRPα	Radiotherapy (RT) promotes CD47 expression in an HER2-dependent manner via the NFκB signal pathway at the transcription level.	Breast cancer	Dual blockade of CD47 and HER2 enhances the antitumor efficacy of RT	[59]
	Low-dose RT increases expression of CD47 on tumor cells via possibly JAK2/STAT3 pathway at the transcription level.	Non-small cell lung cancer (NSCLC)	Low-dose irradiation combined with CD47 inhibitors could inhibit tumor growth.	[168]
	RT can upregulate CD47 through a DNA damage response pathway by ATR-Chk1-STAT3 signaling at the transcription level.	Colorectal cancer	RT combined with anti-SIRPα has effective antitumor effects in both irradiated and abscopal tumors	[60]
	RT-induced upregulation of CD47 is positively associated with DNA damage response genes, such as mer-11, γH2AX, RAD50, NBN, ATM, and ATR at the transcriptional and translational levels.	Mesothelioma	A combination of anti-CD47 immunotherapy and RT is a novel strategy to inhibit tumors.	[169]
	RT-induced fatty acids oxidation (FAO) metabolic signal can increase CD47 transcription by increasing NF-κB/RelA acetylation.	Glioblastoma	Targeting FAO-CD47 axis may improve tumor control in GBM radioimmunotherapy.	[58]
	Radiotherapy combined with glutamine blockade upregulates CD47 expression, increases infiltration of M2 macrophages and inhibits the phagocytosis function of macrophages	Head and neck squamous cell carcinoma (HNSC)	Glutamine and CD47 are potential targets in sensitizing radiotherapy and anti-phagocytic cancer cells.	[147]
	The tristetraprolin (TTP) suppressed by DNMT1‑mediated DNA methylation (Me) upregulates CD47 expression by impairing CD47 mRNA degradation at the post‑transcriptional level by binding to 3’UTR of CD47 AU‑rich element (ARE) 9.	Head and neck cancer	The TPP could become a novel biomarker to predict the efficacy of CD47 antibody in radiotherapy.	[57]
	RT-induced expression change of CD47 depends on post-radiation time points in which radiotherapy inhibits CD47 mRNA expression at 2 h, 6 h,12 h, and 24 h through radiotherapy-induced hsa-miR-222 increasing to bind with 3’ UTR of CD47 by p-ERK signaling but upregulates at 48 h.	Cervical cancer and NSCLC	The miR-222 enhances cancer cell tumor cells sensitivity to radiotherapy by the CD47-pERK pathway in tumor patients.	[65]
	RT upregulates CD47 transcriptional expression by promoting histone H3 lysine 4 methylation (H3K4me) and histone H3 lysine 27 acetylation (H3K27ac) on CD47 promoter.	Glioma	Targeting H3K4me-CD47 might be a potential strategy to sensitize tumors to RT.	[66]
	RT-induced AREG promotes CD47 expression by activating STAT3.	NSCLC	Targeting AREG might inhibit tumor metastasis in radiotherapy combined with anti-CD47 immunotherapy.	[63]
	RT promotes CD47 expression but no detailed mechanisms are available.	Colon cancer, Colorectal cancer, Glioblastoma	RT combined with anti-CD47 is a promising antitumor strategy.	[61, 62, 170]
**PD-L1-PD-1**	RT-induced DNA double-strand breaks (DSBs) and depletion of DSBs repair-associated factors BRCA2, PALB2 and Ku70/80 complex upregulate PD-L1 by activating ATM/ATR/Chk1 – JAKs/ STATs/ IRF1 signaling axis.	Osteosarcoma	BRCA2, PALB2 and Ku70/80 could be new markers to predict the efficacy of anti-PD-1 combined with radiotherapy.	[88]
	RT-induced upregulate of PD-L1 via Karyopherin-α (KPNA) and karyopherin-β1 (KPNB1)-interferon regulatory factor 1 (IRF1) signal pathway.	HNSC, NSCLC	KPNA2 may be a potential target for improving antitumor effect of RT.	[171]
	RT-induced ATR-CHK1-STAT3 DSBs signal pathway upregulates the expression of PD-L1.	Colorectal cancer	RT/anti-SIRPα/anti-PD-1 is a promising antitumor approach in primary tumor and metastatic tumor.	[60]
	RT promotes PD-L1 expression by bromodomain containing 4 (BRD4)-IRF1 axis.	NSCLC	Targeting BRD4 could augment antitumor immunity of RT and anti-PD-1	[172]
	RT increases the tyrosine phosphatase of SHP-2 in PD-1 cytoplasmic domain in M1 TAMs.	NSCLC	The SHP-2 inhibition could further enhance antitumor effects of RT and anti-PDL1.	[87]
	RT upregulates PD-L1 via cGAS-STING-TBK1-IRF3 signaling pathway.	Hepatocellular carcinoma	Targeting cGAS-STING pathway could promote immune effects of RT.	[89]
	Low-dose fractionated radiotherapy upregulates PD-L1 by depending immune molecular IFN-γ.	Colon carcinoma	PD-L1 blockade could overcome radioresistance.	[90]
	RT upregulates PD-L1 via activating epidermal growth factor receptor (EGFR)-JAK2 pathway.	Glioma	RT combined with anti-PD-1/PD-L1 and EGFR-TKIs might be a potential antitumor strategy.	[91]
	RT increases PD-L1 expression via JAK/Stat1 signaling.	Pancreatic ductal adenocarcinoma	Anti-PD-L1 could enhance tumor cells sensitivity to radiotherapy.	[173]
	Diverse RT schemes (18x2Gy, 3x8Gy and 1×16.4 Gy) promote expression of PD-L1 after post-irradiation 7 days.	Colon cancer	The 3×8 Gy could be the most effective RT scheme when associated with anti-PD-L1.	[174]
MHC-1-LILRB1	RT increases the expression of MHC class I and its component β2-microglobulin in a radiation dose-dependent manner.	Glioma	RT combined with tumor vaccination with high expression of MHC-1 might enhance survival rates.	[175]
	RT can upregulate MHC-I on the surface of Ewing’s sarcoma cells subpopulation with low-level expression of APLP2.	Ewing’s sarcoma	Targeting APLP2/MHC-I interactions may boost the antitumor effects of RT.	[176]
	RT upregulates MHC-I at different time points with different mechanisms (early effects: old protein degradation-derived peptides; late effects: new protein-derived peptides) by activating mTOR pathway.	Colon adenocarcinoma	RT can improve the efficacy of tumor immunotherapy	[3]
	RT upregulates MHC-I at transcriptional level by increasing MHC-I transactivator NLRC5.	Pancreatic adenocarcinoma	Targeting NLRC5 might improve antitumor effects of RT combined with immunotherapy.	[177]
	RT upregulates MHC-I by NF-kB/IFN-β signaling axis.	Breast cancer	Combination of RT and immunotherapy might activate a strong immune response to inhibit tumor growth.	[178]
CRT-LRP1	RT-induced ER stress facilitates CRT translocation to the tumor cells surface.	Breast, lung, and prostate cancer	RT combined with immunotherapy might be an effective strategy for patients failing RT alone.	[6, 120]
	Different RT types, including photon, proton and carbon-ion, can increase CRT membrane exposure.	Glioma, lung, tongue squamous and nasopharyngeal cancer	Different RT types (proton, carbon-ion, photon) combined with immunotherapy might be a novel antitumor manner.	[121]
PS-associated axis	RT can upregulate PS on the surface of tumor cells by depending on caspase activity	Pancreatic cancer	Cancers might be grouped into low or high surface PS to predict respectively tumor cells sensitivity to radiotherapy.	[133]

### “Don’t eat me” signals

#### CD47-SIRPα axis

##### Immune-related characteristics of the CD47/SIRPα axis

The CD47 protein is universally expressed on cancer cells [46, 47]. CD47 conveys inhibitory “don’t eat me” signals to its receptor SIRPα on the phagocytes (such as macrophages, DCs [47], neutrophils [48]) to activate inhibitory anti-phagocytosis signals [49, 50]. Various transcription factors can upregulate CD47 on the surface of cancer cells, such as MYC [51], nuclear factor kappa B (NFκB) [52], signal transducer and activators of transcription 3 (STAT3) [53], and hypoxia-inducible factor-1(HIF-1) [54]. Targeting CD47/SIRPα not only enhances the innate response through macrophage-mediated phagocytosis [48, 55] but also promotes adaptive immunity, which activates antitumor-specific T cells [56]. Therefore, CD47/SIRPα-targeted therapy is a promising antitumor therapy [47, 48].

##### Radiotherapy effects on the CD47/SIRPα axis

The CD47/SIRPα axis is usually overexpressed in diverse radioresistant tumors, such as head and neck cancer [57], glioblastoma multiforme [58], and breast cancer cells [59]. Radiotherapy mainly upregulates CD47 [59–61] and SIRPα [62] in the most diverse tumor microenvironments. For example, radiotherapy upregulates CD47 on tumor cells [61] and SIRPα expression on myeloid cells in colorectal cancer [62]. Radiotherapy-induced upregulation of amphiregulin (AREG) not only promotes CD47 upregulation via STAT3 activation in tumor cells but also reprograms EGFR+ mononuclear phagocytes into an immunosuppressive phenotype, resulting in impaired mononuclear phagocyte phagocytosis [63]. However, radiotherapy downregulates CD47 in oropharyngeal squamous cell carcinoma (OSSC), resulting in increased phagocytosis by dendritic cells (DCs) [64]. The molecular mechanisms underlying radiotherapy-induced CD47/SIRPα expression changes have been at least partly elucidated in different tumor types and are summarized in Table 1.

##### The immune responses after radiotherapy-induced CD47/SIRPα expression

Radiotherapy-induced effects are mainly related to radioresistance by upregulation of CD47 or SIRPα, but other studies [64, 65] demonstrated that it can also induce tumor cells sensitivity to radiotherapy by inhibiting CD47 expression. For example, radiotherapy-induced upregulation of CD47 inhibits phagocytosis function of macrophages, promotes M2 polarization of macrophages and inhibits activation of CD8 + T cells to induce radioresistance of GSCs [66]. However, radiotherapy-induced microRNA can inhibit CD47 expression to promote tumor cells sensitivity to radiotherapy in cervical carcinoma, kidney carcinoma and human alveolar adenocarcinoma [65]. Therefore, radiotherapy combined with CD47/SIRPα axis-associated immunotherapy can further promote anti-tumor efficiency, which is mainly associated with reshaped immune-activated irradiated tumor microenvironments (Table 2).

**Table 2:** The anti-tumor immune responses after radiotherapy and phagocytosis checkpoints-associated immunotherapy.

Signaling pathway	Style of therapy	Innate immune response	Adaptive immune response	Abscopal effect	Ref.
CD47-SIRPα	Single high-dose RT + anti-CD47	Increased M1 macrophages Decreased M2 macrophages, MDSCs	Increased CD8+ T cells and IFN-γ+ T cells	/	[61]
	Hypo-fractionated RT + anti-SIRPα +anti-PD-1	Increased M1 macrophages, DCs Decreased M2 macrophages, MDSCs	Increased IFN-γ and TNF-α CD8+ T cells	/	[62]
	RT + anti-CD47	Increased SIRPα-deficient macrophages Decreased MDSCs	Increased CD8+ T cells, NK cells and inflammatory neutrophils Decreased regulatory T cells	/	[179]
	RT + anti-CD47	Increased pro-phagocytic signals	/	/	[180]
	Low-dose RT + anti-CD47	/	Increased CD8+ T cells	/	[168]
	RT+anti-CD47	/	Increased granzyme B expression in CD8+ T cells	/	[37]
	RT + anti-CD47	RT-induced tumor-infiltrating macrophages and monocytic MDSCs promote phagocytosis.	Increased CD8+ T cells	/	[38]
	Radiotherapy	Increased phagocytosis of bone marrow-derived dendritic cells	Increased IFN-γ and granzyme B production from mixed lymphocytes	/	[64]
	RT+anti-CD47	Impaired anti-tumor phagocytic activity efficiency	/	/	[181]
	RT + anti-SIRPα+anti-PD-1	Increased phagocytosis by bone marrow-derived DCs	Increased CD8+ T cell cross-priming	Non-irradiated colorectal cancer	[60]
	RT+anti-CD47+anti-PD-1	Increased macrophages	CD8+ T-independent	Non-irradiated small cell lung cancer	[67]
	RT+anti-CD47+anti-HER2	Increased macrophage-mediated phagocytosis	/	/	[59]
	RT+anti-CTLA4+anti-CD47	/	Increased CD3+ and CD8+ T cells and NK cells	/	[182]
PD-L1-PD-1	RT+anti-PD-L1+anti-SHP-2	Increased M1/M2 ratio	Increased CD8+Tcells Decreased Tregs	Non-irradiated small cell lung cancer	[87]
	RT+ anti-PD-L1/anti-PD-1	/	Increased CD8+ T cells	/	[183]
	RT+anti-PD-L1	Increased macrophage infiltration and macrophages phagocytic activity	/	Non-irradiated glioblastoma	[184]
CD24-Siglec10	RT + CD24/Siglec-10 blocked peptide	Increased the phagocytosis, M1/M2 ratio and MDSCs	Increased IFN-γ + CD8+ T cells	/	[106]
CRT-LRP1	RT+anti-PD-L1+caspase-8 knockout	Improving phagocytosis function and antigen presentation of DCs	Increased CD8+ T cells	/	[123]
	RT+ a CRT-coated compound	/	Increased fractions of cytotoxic T lymphocytes, CD4+ T cells, CD8+ T cells and memory T cells	/	[185]
PS-associated axis	RT + anti-PS	Increased M1 macrophages	Increased CD8+ T cells	/	[134]

##### CD47/SIRPα-induced abscopal effects after radiotherapy

Furthermore, the anti–CD47/SIRPα axis not only inhibits local irradiated tumor cells but also inhibits distant non-irradiated tumor cells [60, 67, 68]. CD47 blockade combined with radiotherapy promotes abscopal effects to inhibit distant, non-irradiated small cell lung cancer. The radiotherapy-induced secretion of cytokines from tumor cells, such as CSF1, CCL2, and MCP3, promotes macrophage recruitment and activation from the irradiated tumor microenvironment to the non-irradiated tumor site [67]. Therefore, CD47/SIRPα is a novel mechanism of abscopal response independent of CD8+ T cells [68]. Previous studies have shown that the abscopal effects are mainly associated with tumor-draining lymph nodes(TDLN) [69] and CD8+ T cells [70] because radiotherapy could impair T cells in these TDLN. For example, elective nodal irradiation(ENI) inhibits abscopal responses by decreasing active CD8+ T cells in non-irradiated head and neck tumors [71]., whereas the delayed TDLN irradiation has more effective antitumor effects in metastatic melanoma compared to simultaneous radiotherapy for lymph nodes and tumors [72]. However, in clinical practice, it is difficult to distinguish which TDLNs have not metastasized and therefore avoid their irradiation to retain function of T cells. Therefore, macrophages, as the immune cells with a relatively large residual amount after radiotherapy, can compensate for the weakened abscopal effect caused by the reduction of T cells due to lymph node injury after radiotherapy.

##### Anti-tumor effects with phagocytosis-independent mechanism in radiotherapy

Beyond phagocytosis-related antitumor immune response, the anti-CD47/SIRPα axis also enhances tumor cells sensitivity to radiotherapy through phagocytosis-independent mechanisms. Anti-CD47 enhanced tumor cells sensitivity to radiotherapy in oral squamous cell carcinoma (OSCC) by suppressing cancer stem cell-like phenotype [73]. Also, anti-CD47 can enhance tumor cells sensitivity to radiotherapy by inhibiting tumor pluripotency capabilities and reducing the expression of DNA repair enzymes in OSCC [74].

##### Radioprotection effects of CD47 blockade in normal cells

However, some studies indicated that CD47 blockade can have different radiotherapy effects between normal and tumor cells [75, 76]. Blocking CD47 combined with radiotherapy has radioprotection effects in normal tissues and organs, which contribute to normal cell survival involving various tissues (muscle, skin, vascular, endothelial tissue) and bone marrow [77, 78], whereas it increases tumor cells sensitivity to radiotherapy [79]. The main mechanism underlying such selective radiotherapy reaction is that CD47 not only serves as a receptor of SIRPα on phagocytosis but also as a receptor of the secreted matricellular glycoprotein thrombospondin-1(TSP-1) [80]. Therefore, radiotherapy combined with anti-CD47 not only promotes tumor cells sensitivity to radiotherapy but also has radioprotective effects on normal tissues and organs.

#### PD-L1/PD-1 axis

##### Immune-related characteristics of PD-L1/PD-1 axis

The PD-L1/PD-1 interaction between tumor cell and T cell contributes to escape from T cell-mediated tumor immune surveillance by acting as a “don’t find me” signal [81, 82]. However, PD-L1/PD-1 axis is also a phagocytosis checkpoint. PD-L1 is expressed on tumor cells, and its receptor PD-1, which is an inhibitory transmembrane protein, is expressed on phagocytes to activate anti-phagocytosis signals [40, 83]. PD-1(+) tumor-associated macrophages (TAMs) have lower phagocytosis function compared to PD-1(-) TAMs and PD-L1 knockout promotes phagocytosis function of PD-1(+) macrophages [40]. High expression of PD1 on macrophages predicts poor outcomes [84]. Therefore, PD-L1/PD-1 blockade not only improves adaptive immune responses associated with activated T cells but also promotes innate immune responses associated with macrophages phagocytosis.

##### Radiotherapy effects on the PD-L1/PD-1 axis

In radioresistant head and neck squamous cell carcinoma cells, PD-L1 expression is upregulated in nuclear and cytoplasmic cell fractions [85]. Also, high PD-L1 expression is positively correlated with radioresistance in NSCLC [86]. Furthermore, radiotherapy increases the tyrosine phosphatase SHP-2 of PD-1 cytoplasmic domain in the M1 TAMs in NSCLC [87]. The radiotherapy-induced mechanisms of PD-L1 expression are mainly associated with DNA damage and repair signaling pathway [88], cGAS-STING pathway [89], IFN-γ signaling [90] and epidermal growth factor receptor (EGFR) pathway [91]. However, most researchers mainly study how radiotherapy-induced PD-L1/PD-1 expression changes influence adaptive antitumor immune responses mediated by CD8+ T cells but not innate immune responses mediated by phagocytosis.

##### The immune responses after radiotherapy combined with PD-L1/PD-1 blockade

There is great research associated with radiotherapy combined with PD-L1/PD-1 blockade to activate antitumor CD8+ T cells [92, 93]. However, innate antitumor immune responses also may play a significant role in the combination treatment. Radiotherapy combined with anti-PD-1 promotes tumor cell phagocytosis by DCs, increases tumor-associated antigen presentation, and further promotes tumor-specific CD8+ T cells priming in colorectal cancer. Furthermore, radiotherapy combined with anti-PD-1 also increases abscopal effects [60]. Anyway, although the anti-PD-L1/PD-1 axis as “don’t find me” signal in combination with radiotherapy has been studied extensively, its role as a phagocytosis checkpoint combined with radiotherapy needs to be further elucidated (Table 2).

#### MHC-1-LILRB1 axis

##### Immune-related characteristics of the MHC-1-LILRB1 axis

MHC-1 is a transmembrane polymorphic glycoprotein which can process and present tumor antigen fragments to TCR on the surface of CD8+ T cells [94]. However, MHC-I is also expressed on the surface of various cancer cells and its component β2M binds with its receptor LILRB1 on macrophages to inhibit phagocytosis function [41] by activating four ITIM sequences [95]. LILRB1 antibody blocks the interaction of MHC-I and LILRB1 to increase the phagocytosis capability of macrophages, increase M1/M2 ratio and also improve the cytotoxic capability of both NK and T cells to inhibit tumor growth [96]. Therefore, anti-MHC-I/LILRB1 axis is a promising immunotherapy strategy, which is associated with the phagocytosis function of macrophages [41].

##### Radiotherapy effects on the MHC-1-LILRB1 axis

Radiotherapy can upregulate MHC-I on the surface of various cancers, including glioblastoma [97, 98], cervical cancer [99], colon, lung, prostate cancer [100], ovarian carcinoma [101], melanoma [3] and tongue and mobile tongue squamous cell carcinoma [102]. However, many studies mainly focus on its antigen presentation function to cytotoxic T cells to augment the anti-tumor adaptive immune response but do not mention its negative effects on phagocytosis function.

#### CD24-Siglec-10 axis

##### Immune-related characteristics of the CD24-Siglec-10 axis

CD24, as a cancer stem cell marker [103], has recently been confirmed to be a phagocytosis checkpoint and highly expressed on tumor cells. CD24 interacts with its inhibitory transmembrane protein receptor Siglec-10 on macrophages, resulting in the activation of an inhibitory phagocytosis signaling cascade [42, 104]. High expression of CD24 is associated with short survival and both anti-CD24 on tumor cells and anti-Siglec-10 on tumor-associated macrophages effectively promote tumor cell phagocytosis by macrophages [42]. Furthermore, high expression of CD24 is correlated with more lymph node metastases, more advanced pathological stage, and shorter survival in breast cancer [105]. Therefore, targeting CD24/Siglec-10 axis can improve prognosis of cancer patients both as anti-CD24/Siglec-10 monotherapy or in combination with other therapy approaches.

##### Radiotherapy effects on the CD24-Siglec-10 axis

The effect of radiotherapy on CD24/Siglec-10 expression is scarcely known, according to current research. However, Shen, W. et al. indicated that radiotherapy combined with CD24/Siglec-10 blockade has a synergistic anti-tumor efficiency compared with their treatment as single agents by increasing the percentage of IFNγ-expressing CD8+ T cells, ratio of M1/M2 macrophages, and proportion of monocytic MDSCs in colon cancer [106] (Table 2).

##### CD24-Siglec-10 axis as cancer stem cell marker in radiotherapy

While high expression of CD24 is associated with tumor cells sensitivity to radiotherapy because CD24(-)/CD44(+) is a tumor stem marker, its low expression or loss in stem-like breast cancer generates radioresistance by inhibiting radiotherapy-induced tumor cell death, because loss of CD24 leads to low level of radiation-induced ROS and decreased genomic instability [107].

#### Other anti-phagocytosis checkpoints

##### α2-6-linked sialic acid-CD22 axis

CD22 (siglec-2) is also an anti-phagocytosis molecule expressed on the surface of phagocytes but traditionally expressed on B-cells to inhibit B-cell receptor signaling [108]. Pluvinage, J. V. et al. demonstrated that CD22 is upregulated on aged microglia and CD22 binding with *α* 2,6-linked sialic acid inhibits the phagocytic capacity of microglia by activating CD22 downstream inhibitory SHP-1 signaling [43].

##### Fc-FcγR IIB axis

Fc receptors are a series of classical and important phagocytosis-related cell surface receptors expressed on the macrophages, which mediate both anti-phagocytosis and pro-phagocytosis processing by interacting with their ligand IgG immune complexes, especially type I Fc common gamma receptors(FcγR) [109, 110]. In these Fcγ receptors, FcγRIIB mediates anti-phagocytosis on the surface of macrophages to activate inhibitory phagocytosis signals [111, 112].

##### SLAMF3/SLAMF4

Li, D. et al. demonstrated that signaling lymphocytic activation molecule (SLAM) family receptors, particularly SLAMF3 (CD229) and SLAMF4 (CD244), are also “don’t eat me” receptors on macrophages. They confirmed these receptors decrease the phagocytosis function of macrophages by inhibiting low density lipoprotein receptor-related protein 1(LRP1)-mediated activation of mTOR and Syk signaling to inhibit “eat me” signals [44].

##### GD2-Siglec-7 axis

The disialoganglioside GD2 is a sialic acid-linked glycolipid and is widely expressed on diverse tumor cells, especially neuroblastoma [113]. Recently, Theruvath, J. et al. demonstrated that combination treatment of anti-GD2 and anti-CD47 has potent antitumor synergy by promoting macrophages to phagocytose tumor cells in neuroblastoma, osteosarcoma and small-cell lung cancer. Siglec-7 is the ligand for GD2 to mediate “don’t eat me” signals. Interestingly, anti-GD2 not only inhibits anti-phagocytosis signals but also promotes “eat me” signals by upregulating surface calreticulin on tumor cells [45].

However, the above-mentioned anti-phagocytosis checkpoints have not been studied with radiotherapy according to current research.

### “Eat me” signals

#### Calreticulin (CRT)-LRP1 axis

##### Immune-related characteristics of CRT-LRP1 axis

Calreticulin(CRT) is a multifunctional protein in the endoplasmic reticulum(ER) [114]. Under ER stress induced by chemotherapy [115] and radiotherapy [7], CRT can translocate from the lumen of ER to the surface of tumor cells. The low-density lipoprotein receptor-related protein 1 (LRP1, CD91) [116] is the ligand for CRT and is expressed on the surface of phagocytes to promote phagocytosis of tumor cells [114]. Chao, M. P. et al. suggested that CRT is highly expressed both on hematologic malignancies and solid tumors whereas it is less expressed on normal cells. Furthermore, high CRT expression is associated with increased CD47 expression and CRT-LRP1 blockage abrogates anti-CD47 antibody-mediated phagocytosis [117]. Different molecular mechanisms, including stanniocalcin1 (STC1) [118] and hormone glucocorticoid (GC) [119] regulate CRT and LRP1 expression.

##### Radiotherapy effects on the CRT-LRP1 axis

Radiotherapy is also a regulator of CRT-LRP1 signal axis for which radiation-induced ER stress facilitates CRT translocation to the tumor cells surface, resulting in promoting immunogenic cell death by increasing antigen-specific CD8+ cytotoxic T lymphocytes [6, 120]. Radiotherapy upregulates CRT in cervical cancer patients’ tumor biopsy specimens [39]. Moreover, different radiotherapy types, including photon, proton, and carbon-ion, all can increase CRT membrane exposure in lung adenocarcinoma, glioma, tongue squamous carcinoma, and nasopharyngeal carcinoma in a dose-dependent manner [121]. These different radiotherapy types can upregulate CRT under normoxic conditions, especially carbon-ion radiation. However, under hypoxic conditions, the baseline expression level of CRT is high enough, and radiotherapy could not further increase the expression of CRT [122].

##### The immune responses after radiotherapy combined with the CRT-LRP1 targeting therapy

Radiotherapy-induced CRT upregulation can improve antitumor effects of anti-PD-L1 treatment in caspase-8 knockout tumors because the knockout of caspase-8 suppresses the translocation of CRT to the surface of tumor cells, which impairs phagocytosis function and antigen presentation of DCs and the infiltration of tumor-specific CD8+ T cells [123]. Furthermore, radiotherapy-induced upregulation of CRT inhibits tumor neurospheroid formation and tumor stemness to increase tumor cells sensitivity to radiotherapy in neuroblastoma [124] (Table 2).

##### The negative role of CRT-LRP1 axis in tumors

Interestingly, high expression of CRT is associated with worse clinical outcomes in different tumors [117]. Also, LRP1 is highly expressed in radioresistant colorectal cancer and higher expression of LRP1 is associated with poor clinical outcomes [125]. The high expression of mutated CRT possibly can explain this interesting phenomenon. Soluble exon-9-mutated CRT is highly released from ER of tumor cells to further inhibit phagocytosis by DCs and induce immunosuppressive effects in osteosarcoma, cervix adenocarcinoma, fibrosarcoma and NSCLC [126].

#### Phosphatidylserine (PS)-TIM4/CD300b/BAI1/ Stabilin-2

##### Immune-related characteristics of PS-associated axis

Phosphatidylserine (PS), as an “eat me” signal, is an inner cell membrane molecule at normal physiological environment but can also translocate to the surface of apoptotic cells under different molecular signals [127–129]. PS has different receptors [130] on the surface of phagocytes to promote phagocytosis, such as T-cell immunoglobulin mucin-4 (TIM4), single Ig-domain type I transmembrane protein CD300b, brain-specific angiogenesis inhibitor 1 (BAI1), Rage, Scarf1, CD36, Trem2 and transmembrane protein Stabilin-2 [130–132].

##### The effects of radiotherapy on PS-associated axis

Radiotherapy can upregulate PS on tumor cells by inducing caspase activity, and high expression of PS is associated with radioresistance [133]. However, radiotherapy-induced PS upregulation promotes immunosuppressive signals and targeting PS antibody combination with radiotherapy can enhance anti-tumor efficiency in melanoma by increasing M1 phenotype macrophages and tumor antigen-specific CD8+ T cells [134]. However, it is needed to further study the influence of radiotherapy-induced PS upregulation on phagocytosis function in future studies.

#### Other pro-phagocytosis axis

##### SLAMF7-SLAMF7 axis

SLAMF7 is expressed exclusively on the surface of hematologic tumor cells, which promotes tumor cells phagocytosis by binding with its equal receptor SLAMF7 on phagocytes [135]. Chen, J. et al. demonstrate that SLAMF7 on macrophages promotes phagocytosis by increasing polarization of actin associated with key step of phagocytosis process [135]. Interestingly, another study suggested that SLAMF7 can express highly on solid breast cancer, and high SLAMF7 expression is associated with poor clinical outcomes, for which SLAMF7 mediates “don’t eat me” signals [136].

##### Fc-FcγRs axis

FcγRI, FcγRIIB, FcγRIII, and FcγRIV on macrophages mediates pro-phagocytosis signals by binding with IgG Fc domain of target cells [110, 137]. The binding of Fc and pro-phagocytosis FcγRs also promotes phosphorylation of ITAMs by activating tyrosine kinase and further increases downstream phagocytosis signals in macrophages [138].

Unfortunately, there are no studies on revealing the effects of radiotherapy on the expression of SLAMF7 and Fc-FcγRs axis according to present literature.

## Clinical application of phagocytosis checkpoints in tumor radiotherapy

**Fig. 3:**
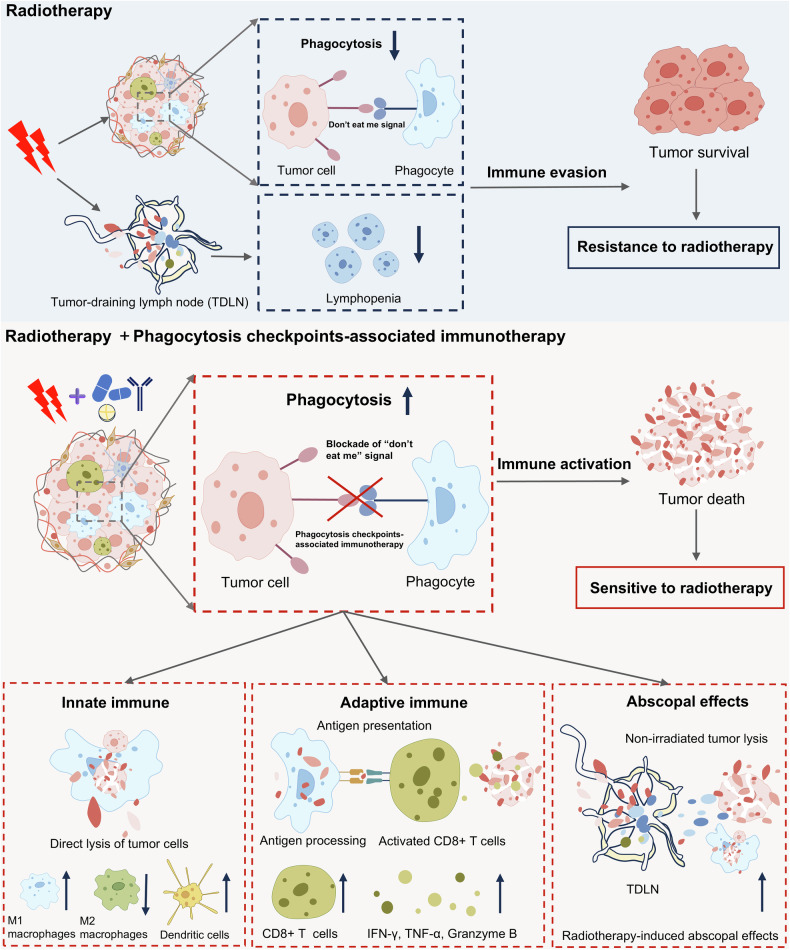
The phagocytosis checkpoint-associated immunotherapy converts radioresistant tumors into radiosensitive ones. Radiotherapy-induced reduction of phagocytosis function and lymphopenia promote tumor immune evasion leading to radioresistance. Combination of radiotherapy and phagocytosis checkpoints-associated immunotherapy could convert radiotherapy-resistant tumors into radiotherapy-sensitive ones by promoting phagocytosis function and further activating antitumor immune response. In the combination treatment, innate immunity could be enhanced by directly lysing tumor cells by increasing infiltration of M1 macrophages and dendritic cells but decreasing infiltration of M2 macrophages. Also, phagocytes as antigen presentation cells could process and present tumor antigen to increased CD8+ T cells and further promote the release of IFN-γ, TNF-α and granzyme B from activated CD8+ T cells. Furthermore, the combination treatment could enhance abscopal effects in non-irradiated tumor sites through more macrophages remaining after radiotherapy in tumor-draining lymph nodes. (Created with Microsoft Office PowerPoint).

**Fig. 4:**
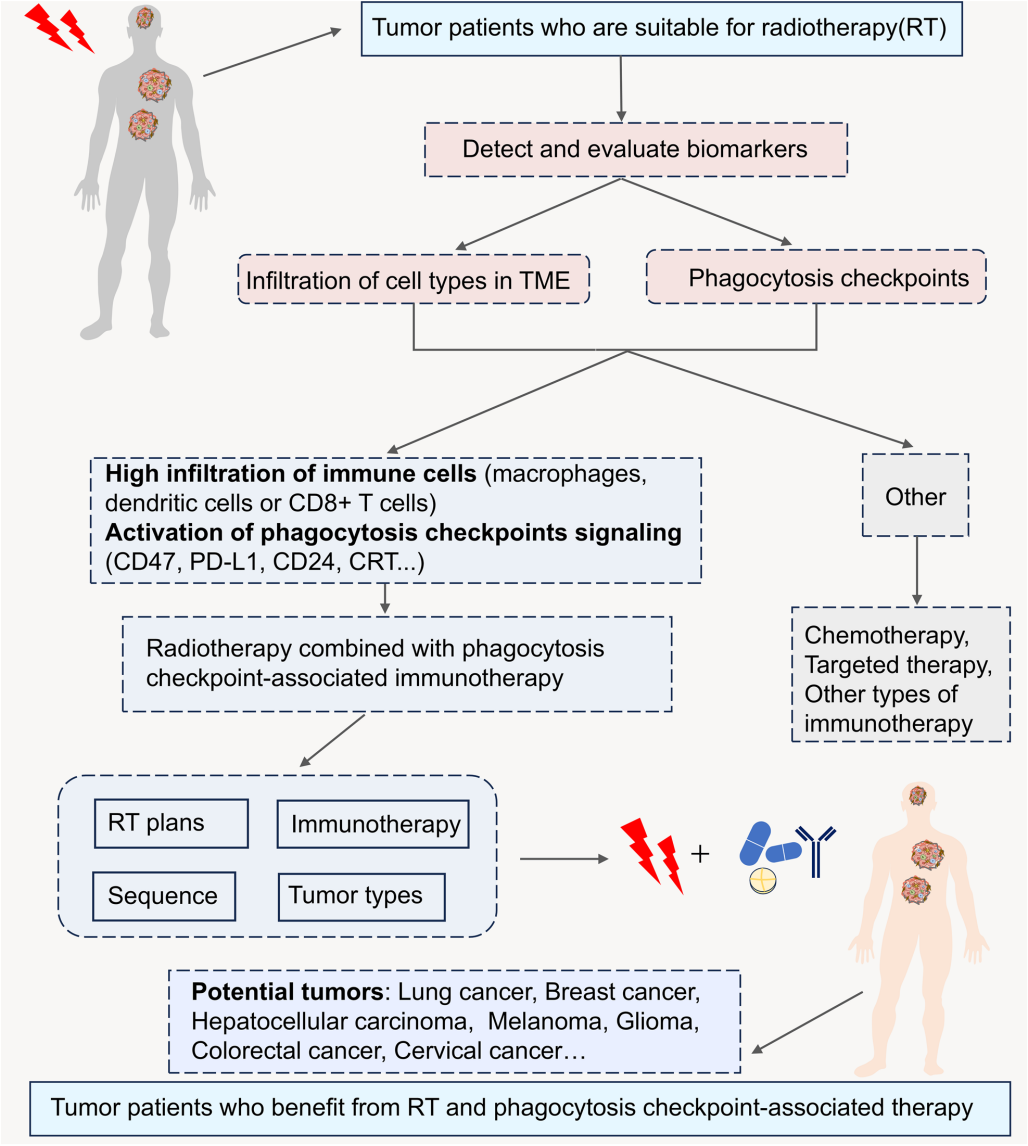
Clinical application of phagocytosis checkpoints-associated immunotherapy in radiotherapy by detecting and evaluating phagocytosis checkpoints-related biomarkers. Detecting and evaluating biomarkers is a wise choice for the combination of radiotherapy and immunotherapy to select an appropriate immunotherapy regimen for tumor patients who are suitable for radiotherapy. Given that radiotherapy regulates phagocytosis checkpoints, it is a wise choice to select phagocytosis checkpoints as new biomarkers in combination with radiotherapy and immunotherapy. Patients with high infiltration of antitumor immune cells (such as macrophages, DCs, CD8+ T cells) or high activation of phagocytosis checkpoints (such as CD47, PD-L1, CD24, CRT) could be selected to the combination of radiotherapy and phagocytosis checkpoints-associated immunotherapy. In the combination treatment, it is needed to consider radiotherapy plans, immunotherapy schemes, sequence of combination treatment and tumor types for enhancing antitumor effects of the novel combination treatment. According to current studies, tumor patients with lung cancer, breast cancer, liver cancer, melanoma, glioma, colorectal cancer or cervical cancer might acquire benefits from the novel combination treatment. However, for patients with low infiltration of immune cells or low activation of phagocytosis checkpoints, other treatments need to be selected, such as chemotherapy, targeted therapy and other types of immunotherapy and so on. (Created with Microsoft Office PowerPoint).

Given that numerous preclinical studies as previously described, the potential combination treatment of radiotherapy and phagocytosis checkpoints-associated immunotherapy is a promising treatment strategy for cancer patients (Fig. 3). Besides, these phagocytosis checkpoints molecules have other functions which make the novel combination treatment have stronger anti-tumor effects compared to T cell-mediated immune checkpoint-related immunotherapy (Table 3). In clinical applications, phagocytosis checkpoints can be putative biomarkers to predict tumor cells sensitivity to radiotherapy to realize individualized treatment but not blind treatment (Fig. 4). Also, targeting these molecules in radiotherapy would have synergistic efficiency by using feasible and optimal therapy strategies.

**Table 3:** Phagocytosis checkpoint characteristics in tumor immune microenvironment.

Signaling pathway		RT-associated pre-clinical study	Other functions	Radiotherapy efficacy (Activated signaling)	Prognostic significance (Activated signaling)
“Don’t eat me” signals	CD47-SIRPα	**√** (Identified)	Radioprotection in normal cells [77, 78]	Radioresisatnce [58]	Negative [48]
	PD-L1-PD-1	**√**	T cell-mediated tumor adaptive immune [18]	Radioresistance [85]	Negative [83]
	MHC-1-LIRB1	**√**	Processing and presenting tumor antigen [94]	Sensitivity to radiotherapy [186]	Negative [41] Positive [187]
	CD24-Siglec10	**√**	Cancer stem cell marker [103]	Radioresistance [188]	Negative [189]
	α2–6-linked sialic acid -CD22	/ (Not identified)	Inhibiting B-cell receptor signaling [108]	/	Negative [190]
	Fc-FcγR IIB	/	/	/	Negative [112] Positive [191]
	SLAMF3/SLAMF4	/	CD8+ T cells exhaustion [192]	/	Negative [44] Positive [193]
	GD-2-Siglec7	/	Inhibiting CD8+ T cells [194]	/	Negative [113]
“Eat me” signals	CRT-LRP1	**√**	Activating DCs [195]	Sensitivity to radiotherapy [124]	Negative [196] Positive [197]
	PS-associated axis	**√**	Promoting immune suppression [198]	Radioresistance [133]	Negative [199]
	SLAMF7-SLAMF7	/	Activating CD8+ T cells [200]	/	Positive [201]
	Fc-FcγRs	/	/	/	Negative [112] Positive [191]

### Phagocytosis checkpoints as potential biomarkers for prediction of tumor cells sensitivity to radiotherapy

Radiotherapy is not always an effective antitumor manner with low radiotherapy curative effect or radioresistance because of various and complicated tumor and patient characteristics [139]. Therefore, it is necessary to find assessment methods for evaluating whether tumor patients are suitable for radiotherapy. The main assessment method includes biomarkers that can predict the efficacy of radiotherapy in diverse tumors [140–142] (Table 4).

**Table 4:** The potential clinical application of radiotherapy combined with phagocytosis checkpoints-associated immunotherapy.

Types of application		Details of application	Ref.
Biomarkers for sensitivity to radiotherapy	CD47	CD47 has been upregulated in radioresistant breast cancer cells.	[59]
	PD-L1	Overexpression of PD-L1 is a helpful biomarker of radiotherapy treatment failure in HPV-negative head and neck squamous cell carcinoma.	[202]
		Overexpression of PD-L1 is a main marker to radioresistance in lung adenocarcinoma.	[203]
		Low expression of PD-L1 is correlated with radioresistance and poor prognosis in head and neck squamous cell carcinoma.	[204]
		High expression of PD-L1/PD-1 is related to tumor cells sensitivity to radiotherapy in head and neck cancers	[205]
	CD24	The lack of CD24 at the level of primary clonogenic blasts is related to irradiation resistance in B-lineage acute lymphoblastic leukemia patients.	[206]
		The CD24-negative breast cancer stem cells are markers of radioresistance.	[207]
		The CD24-positive pancreatic cancer stem cells are resistant to radiotherapy.	[208]
	CRT	Glioblastoma cells overexpressing CRT have increased tumor cells sensitivity to radiotherapy.	[209]
Diverse radiotherapy (RT) plans	RT dose fraction	High-dose RT (12 Gy) to primary tumor site primes T cells and low dose RT (1 Gy × 2 fractions) to secondary site promotes M1 macrophage polarization and NK cell infiltration.	[210]
		Low-dose RT (2 Gy X 1 fraction) promotes systemic antitumor effects of hypofractionated RT (8 Gy X 3 fractions) combined with anti-PD1 therapy.	[211]
		Both a single-fraction dose of 5 Gy and a fractionated schedule (20 Gy /5 fractions) have the same anti-tumor efficiency when RT is combined with CD47 blockade.	[67]
		Both low-dose fractionated and hypofractionated RT did not enhance progression-free survival and overall survival compared to anti-PD-L1 inhibition alone in metastatic NSCLC.	[33]
	RT types	Proton radiation could upregulate “eat me” signal protein CRT expression on the tumor cells to promote tumor antigen presentation by phagocytes and further increase infiltration of CD8+ T cells.	[212]
		The carbon ion therapy in combination with anti-PD-1 antibody not only can upregulate CRT but also increase infiltration of CD4+ and CD8+ T cells compared to conventional radioimmunotherapy.	[213]
		Other new radiotherapy styles such as spatially fractionated RT and FLASH RT also have antitumor immune responses and can combine with immunotherapy to produce more effective antitumor efficiency.	[214]
Targeting strategies for phagocytosis checkpoints-associated immunotherapy	Antibody	Anti-CD47 antibody Hu5F9-G4 combined with rituximab (anti-CD20) inhibits B-cell non-Hodgkin’s lymphoma progression by increasing macrophage-mediated phagocytosis in a phase 1b study.	[25]
		Anti-CD47 antibody Hu5F9-G4 has a well-tolerated anti-tumor efficiency both in solid tumors and hematologic tumors.	[26]
		Anti-SIRPα antibody is a promising antitumor drug with fewer hematologic toxicities side effects because of the confined expression of SIRPα on normal cells compared with CD47.	[49]
		Anti-SIRPα antibody promotes phagocytosis of macrophages, activation of DCs and further increases cross-priming tumor-specific CD8+ T cells.	[215]
	Small molecule inhibitors	The MYC inhibitor JQ1 downregulates CD47 and PD-L1 expression on the tumor cells to increase antitumor immune response.	[51]
		The QPCTL inhibitor regulates CD47 pyroglutamate formation to interfere the binding with SIRPα promoting tumor cell killing by macrophages and neutrophils.	[216]
		The EGFR inhibitor gefitinib inhibits the expression of CD47 and increases the expression of CRT, which promotes tumor cell phagocytosis by monocyte-derived dendritic cells in human NSCLC.	[217]
	Peptide	A macrocyclic peptide D4-2 can block CD47-SIRPα interaction by selectively binding with g-V-like domain of SIRPα, which further promotes macrophage-mediated phagocytosis.	[218]
		The novel peptide pep-20 combined with RT (a single dose of 20 Gy) blocks the CD47/SIRPα interaction by binding to the CD47-IgV domain and inhibiting SIRPα tyrosine phosphorylation of ITIMs, resulting in promoting macrophage-mediated phagocytosis and activating antitumor T-cell immune response.	[38]
		A peptide named hEL-RS17 could bind to CD47 on tumor cells and block the signaling of CD47-SIRPα.	[219]
	Nanomaterials	A nanobioconjugate engager carrying both anti-HER2 antibodies and CRT to increase the breast cancer cells phagocytosis and tumor antigen presentation by macrophages.	[220]
		An engineered biomimetic nanozyme CD47@CCM-Lap-CuS NP, its near-infrared laser irradiation can generate photothermal therapeutic effects on CD47-overexpressing cancer cells.	[221]
		The integration of nanoscale metal–organic frameworks enabled radiotherapy with checkpoint blockade immunotherapy to have both local and systemic antitumor effects.	[222]
		Additional injection of NBTXR3 nanoparticles can enhance infiltration and activation of cytotoxic immune cells and antitumor effects of the combination of proton therapy and anti-PD-1 on both irradiated and unirradiated tumors.	[223]
		A bridging-lipid nanoparticle(B-LNP) with dual targeting to irradiation-triggered CD47 and PD-L1 promotes macrophages to engulf tumor cells, antigen presentation and T cell recruitment in irradiated glioma.	[170]
	Multiple immunotherapy regimens	The addition of anti-PD-L1 antibody promotes immune response to the treatment resistance of combination therapy of radiotherapy and anti-CTLA4.	[224]
		Combining two types of phagocytosis checkpoint drugs (anti-SIRPα and anti-PD-1) with RT could activate robust adaptive antitumor immune responses in colorectal cancer.	[60]
Sequence of RT and phagocytosis checkpoints-associated immunotherapy	Concurrent therapy	The concurrent administration of anti-PD-L1 and fractionated radiotherapy has a more synergistic antitumor effect compared to sequential administration in colon carcinoma and breast cancer.	[90]
		Patients treated with RT combined with anti-PD-1 within four weeks had better antitumor response than more than four weeks in melanoma brain metastases patients.	[225]
	Sequential therapy	The administration of RT before ipilimumab has better overall survival and less regional recurrence compared with RT after ipilimumab in melanoma brain metastases.	[226]
		Giving RT before immunotherapy has a better overall response rate compared with RT before immunotherapy in 512 patients with cancer metastasis.	[227]
		RT before immunotherapy has superior survival compared with the reverse sequence of therapy in patients with melanoma brain metastases.	[228]
		Giving anti-PD-1 after irradiation can observe abscopal effects but delivering of anti-PD-1 before irradiation inhibits abscopal activity by promoting infiltration of CD8+ T cells in both primary and secondary tumor in colorectal tumor.	[229]
		But, administration of ipilimumab before RT had more effective antitumor efficiency compared with administration of ipilimumab after RT in advanced melanoma patients.	[230]
Patient selection	Biomarkers: There are various biomarkers including molecular genes, immune cells, clinical radiomic models to predict the antitumor efficiency of radioimmunotherapy.		
	Molecular gene biomarkers	A kind of gene signature constituted of six tumor-infiltrating B lymphocyte-specific genes can predict prognosis and response of RT and immunotherapy, which low-risk gene signature group is associated with more immune cell infiltration and better prognosis in lung adenocarcinoma.	[231]
		A radiosensitivity index (RSI) model including 10 genes is a potential biomarker for radioimmunotherapy which low RSI is associated with higher antigen presentation, higher M1 proportion, richer T cell-inflamed activity and IFN-γ response.	[232]
		A PD-L1 tumor proportion score (TPS) ≥ 50% is a biomarker to select patients who can be treated with pembrolizumab and risk-adapted radiotherapy in locally advanced NSCLC.	[233]
	Immune cell biomarkers	Infiltration of CD103+ Tregs and accumulation of lipid metabolism can predict resistance to radioimmunotherapy in glioblastomas.	[234]
		The contents of blood samples including circulating cell-free DNA (cfDNA), CD8 + PD1+/PDL1+ PBMCs and 27 microRNAs are early promising biomarkers to predict the response of RT and immunotherapy in oligoprogressive patients.	[235]
		A special T-cell signature with low CD8+ naive T-cells and high levels of TIM-3 on multiple T-cell populations at baseline is related to good prognosis in metastatic melanoma patients treated with RT and immunotherapy.	[236]
	Clinical radiomic model biomarkers	The CD8 radiomics score is related to progression-free survival, out-of-field abscopal response and overall survival, which can assess tumor heterogeneity and select patients who may benefit from radioimmunotherapy without invasive.	[237]
		The 18F-FDG-PET is a prognostic imaging biomarker which is associated with OS and PFS for patients with recurrent NSCLC by using its metabolic tumor volume (MTV), total lesion glycolysis (TLG) and lean body mass corrected SUV peak (SUL peak).	[238]
		A clinical-radiomic model using XGBoost algorithm can quantitatively predict pathologic complete response of neoadjuvant radioimmunotherapy in esophageal squamous cell carcinoma.	[239]
	AI approach-based biomarkers	A neural network model based on AI approach can simulate tumor growth and treatment response of RT and anti-PD-L1 therapy by integrating pulse interval, radiation dose, drug dose, and timing to study a “causal relationship” and further optimize treatment regimens in radioimmunotherapy.	[240]
	Tumor types: Special tumor types with more macrophage infiltration or high expression of phagocytosis checkpoint molecules might be more sensitive to the novel combination treatment.		
	High infiltration of phagocytes	The tumor types of high infiltration of phagocytes mainly include breast cancer[163], glioma [164], hepatocellular carcinoma [165], colorectal cancer [166], and non-small cell lung cancer [167].	
	High infiltration of CD8+ T cells	The tumor types with high infiltration of CD8+ T cells include melanoma [241], NSCLC [242], colorectal cancer [243], breast cancer [244].	
	High expression of phagocytosis checkpoint molecules	Using tissue microarray (TMA) data indicates that over 60% of patients have high levels of CD47 in ovarian, cervix, gastric, NSCLC, melanoma, glioblastoma multiforme, head and neck cell carcinoma, colon, pancreatic and esophageal cancer and over 40% in hepatocellular carcinoma, urothelial and kidney cancer.	[245]
		Different tumors highly express PD-L1, such as NSCLC, breast, prostate, colorectal, hepatocellular carcinoma, melanoma, gastric, and brain tumors.	[246]
		CD24 is highly expressed on bladder cancer, liver, prostate, ovarian, lung and breast cancer.	[189]
		CRT is highly expressed in triple-negative breast cancer.	[247]

### Potential strategies for targeting the phagocytosis checkpoints combined with radiotherapy

Radiotherapy has different antitumor responses for diverse tumor characteristics. For example, in CURB clinical trial, stereotactic body radiotherapy (SBRT) targeting oligometastatic sites could effectively improve PFS of NSCLC patients after resistance to systemic therapy, but not improve outcomes in breast cancer with oligometastatic sites [143]. Similarly, in EXTEND trials, metastasis-directed therapy via SBRT could also improve PFS in oligometastatic pancreatic ductal adenocarcinoma [144] and prostate cancers [145]. However, nivolumab(anti-PD-1) plus SBRT does not enhance abscopal effects compared to nivolumab alone in patients with metastatic head and neck squamous cell carcinoma [146]. Although SBRT has limited antitumor effects in metastatic breast cancer and HNSCC, radiotherapy plus phagocytosis checkpoints-associated immunotherapy might be an effective therapy because radiotherapy upregulates phagocytosis checkpoints and targeting these molecules could enhance tumor cells sensitivity to radiotherapy in these tumor cells as mentioned in section 2 [59, 147]. Furthermore, we also need to consider the types of tumors that would benefit from the combination of radiotherapy and phagocytosis checkpoints-associated immunotherapy.

However, only two ongoing clinical trials are studying the combination treatment of phagocytosis checkpoint-associated immunotherapy and radiotherapy. One (NCT02890368) is based on the intratumoral injection of TTI-621 (anti-CD47 antibody) combined with different antitumor treatments including radiotherapy in solid tumors. The other one (NCT05967416) is based on the autologous SIRPα-low macrophages (SIRPant-M) administration to confirm the efficiency of SIRPant-M alone or in combination with radiotherapy in relapsed or refractory Non-Hodgkin lymphoma. Although there are few clinical applications in combination therapy, the efficiency of this combination treatment may be improved with different potential strategies by referring to previous immune checkpoint inhibitor treatments in radiotherapy [148] (Table 4).

#### The influence of diverse radiotherapy plans on the novel combination therapy

##### Radiotherapy dose fraction in the novel combination therapy

The radiotherapy dose fraction includes hypo-fractionation (3–20 Gy/fraction), conventional fractionation schemes (1.8–2.2 Gy/fraction) and hyper-fractionation (0.5–2.2 Gy/fraction) [149]. Diverse radiotherapy dose fraction could regulate macrophage-associated innate immune response. The low dose irradiation (2 Gy) promotes the polarization of irradiated tumor-associated macrophages to M1 macrophages in pancreatic cancer [150, 151]. The stereotactic body radiotherapy (SBRT) (6.5 to 7.25 Gy), as a precise, high-dose, hypofractionated radiation treatment technique delivered in few sessions to extracranial targets with maximal tumor control and minimal damage to healthy tissues, activates innate immunity by enhancing proinflammatory M1 macrophages-mediated metabolite elevations of tumor cells in mitigatory prostate cancers [152]. Therefore, in the novel combination treatment, radiotherapy dose fraction may play a crucial role by referring to radioimmunotherapy studies that have been published.

##### Radiotherapy types in the novel combination therapy

At present, the wide application of radiation source types is mainly light photon radiation including X-ray and γ-ray. However, heavy-particle radiation including proton and carbon ion radiation, as new types of radiotherapy, are gradually starting to be applied to tumor treatment with less damage to normal tissues because of their unique Bragg peak [153]. The heavy-particle radiation combination with immunotherapy also has stronger antitumor efficiency compared with light photon radiation.

#### The effects of phagocytosis checkpoints-associated immunotherapy schemes on the combination therapy

##### Immunotherapy types in the novel combination therapy

Given that radiotherapy influences immunity molecule expression, targeting these diverse immune molecules may induce different antitumor efficiency in radiotherapy. A preclinical study indicated that anti-PD-1 or anti-CTLA4 combined with radiotherapy has opposite antitumor effects [154]. Therefore, it is needed to verify and choose which optimal phagocytosis checkpoints-associated immunotherapy scheme is better combined with radiotherapy.

##### Immunotherapy forms in the novel combination therapy

The forms of phagocytosis checkpoints-associated drugs also influence antitumor effects in radiotherapy, such as monoclonal antibodies, small molecule inhibitors, antibody fusion protein and nanoparticles. Targeting the CD47/SIRPα is the most popular phagocytosis checkpoint-associated immunotherapy. For example, anti-CD47 antibody Hu5F9-G4 has a well-tolerated anti-tumor efficiency both in solid tumors and hematologic tumors [26] Also, the anti-SIRPα antibody is a promising antitumor drug with fewer hematologic toxic side effects because of the confined expression of SIRPα on normal cells compared with CD47 [49]. CD47/SIRPα-associated small molecule inhibitors have some advantages including oral administration, shorter half-life, low cost and no immunogenicity compared with CD47/SIRPα antibodies [155]. What’s more, the novel peptide pep-20 also blocks the CD47/SIRPα interaction by binding to the human CD47-IgV domain and inhibiting SIRPα tyrosine phosphorylation of ITIMs. Also, pep-20-D12 in combination with radiotherapy has synergistic antitumor effects [38]. Besides, nanoparticles (gCM-MNs) not only inhibit the CD47-SIRPα axis but also repolarize tumor-associated macrophages to M1 macrophages [156].

##### Multiple immunotherapy regimens in combination with radiotherapy

The three-treatment strategy combining two types of immunotherapy drugs and radiotherapy is also a wise choice for radioimmunotherapy. For example, the application of anti-SIRPα and anti-PD-1 combined with radiotherapy activates more robust adaptive antitumor immune responses in colorectal cancer [60].

#### The sequence of radiotherapy in combination with phagocytosis checkpoints-associated immunotherapy

##### Concurrent therapy in the novel combination therapy

Concurrent therapy is a widely applied combination treatment based on the radiotherapy-induced immune response. However, in the concurrent treatment, toxicity overlap is a big challenge for which both radiotherapy and immunotherapy can produce adverse reactions, and toxicity may increase [157]. Administering radiotherapy immediately after immunotherapy may exacerbate these toxicity events, leading to treatment interruption or dose modification [158]. Hence, when designing treatment plans, it is crucial to arrange the sequence of radiotherapy and immunotherapy reasonably based on the patient’s specific conditions to minimize toxicity risks.

##### Sequential therapy in the novel combination therapy

Likewise, sequential therapy is also a common combination treatment manner, such as radiotherapy before immunotherapy or immunotherapy before radiotherapy. Especially, radiotherapy before immunotherapy is the main order according to current studies for diverse reasons. Firstly, radiotherapy can shift tumor immune microenvironment from an immunosuppressive “cold” tumor state to an immunostimulatory “hot” one by increasing the release of proinflammatory mediators and chemokines and infiltration of immune cells [8, 9]. Therefore, administering radiotherapy first can create a more favorable immune microenvironment for subsequent immunotherapy. Secondly, radiotherapy could induce immunogenic cell death to release tumor-associated antigens [5]. Immunotherapy, in turn, enhances the ability of immune cells to recognize and attack these antigens. Hence, as a first-line treatment, radiotherapy provides more targets for immunotherapy and improves therapeutic outcomes. Thirdly, radiotherapy can upregulate the expression of immune checkpoints on tumor cell surfaces, such as CD47 and PD-L1 [60]. Initiating radiotherapy before immunotherapy enables immunotherapy to work more effectively and inhibits immune evasion. However, the optimal time for immunotherapy after radiotherapy may be in a few days to weeks after radiotherapy (such as 1 to 14 days after radiation therapy) [159–161]. Also, immunotherapy before radiotherapy is another option. If radiotherapy is given first, it may have a certain inhibitory effect on the immune system [13, 15, 16], affecting the safety of subsequent immunotherapy. Immunotherapy could increase tumor-vascular normalization and decrease tumor hypoxia. Hence, if immunotherapy is given first, it could promote subsequent tumor cells sensitivity to radiotherapy [162]. Therefore, when determining the treatment sequence, it is necessary to consider the balance between therapeutic efficacy and toxicity.

#### Biomarkers and patient selection with different tumor types in combination with radiotherapy and phagocytosis checkpoints-associated immunotherapy

##### Biomarkers in the novel combination treatment

A reliable biomarker is essential to select the appropriate patient populations who are suitable for treatment with radiotherapy in combination with phagocytosis checkpoints-associated treatments. There are various biomarkers including molecular genes, immune cells, clinical models, and radiomic models to predict the antitumor efficiency of radioimmunotherapy and help to make optimal clinical decisions in different tumors. However, at present, many types of biomarkers are mainly associated with PD-1/PD-L1 or CTLA4 immunotherapy in radiotherapy, not phagocytosis checkpoint-associated immunotherapy. Therefore, it is urgent to explore novel biomarkers able to predict the antitumor effects of the novel combination.

##### Tumor selection for novel combination therapy

It is important to select appropriate and responsive patients eligible for the combination therapy to maximize the antitumor effects by considering characteristics of tumors and patients. The phagocytosis checkpoints-associated immunotherapy is mainly dependent on phagocytes or expression level of phagocytosis checkpoints molecules to realize its antitumor effects [21]. Therefore, tumor types with more macrophage infiltration and high expression of phagocytosis checkpoint molecules might be more sensitive to the novel combination therapy. These particular tumor types mainly include breast cancer [59, 163], glioma [58, 164], hepatocellular carcinoma [89, 165], colorectal cancer [60, 166], and NSCLC [167, 168]. Furthermore, as summarized in section 2 of this review, there are numerous preclinical studies suggesting that radiotherapy combined with phagocytosis checkpoints-associated immunotherapy has effective antitumor effects in these tumor types.

## Conclusions

Radiotherapy, as a complicated tumor immune effector, is widely applied in combination with immunotherapy. The effects of radiotherapy on phagocytosis checkpoints are an emerging and developing research interest that promises to lead to new immunotherapy in radiotherapy. Moreover, in the irradiated tumor microenvironment, phagocytosis checkpoints not only involve innate immunity to phagocytose tumor cells but also involve the adaptive immune response because macrophages can present tumor antigens to CD8+ T cells to further kill tumor cells. Furthermore, phagocytosis checkpoints also involve systemic abscopal effects by increasing migratory macrophages in the distant, non-irradiated tumor. Therefore, phagocytosis checkpoint molecules can become potential biomarkers or promising targeting immune molecules in radiotherapy to predict or regulate tumor cells sensitivity to radiotherapy and further to enhance the antitumor response of radiotherapy.

Nevertheless, there are still some problems that need to be resolved in this research area, according to current research results. Firstly, the molecular mechanisms of radiotherapy-induced phagocytosis checkpoint expression still need to be further explored, especially other molecule types except for the CD47-SIRPα axis in future studies. Secondly, it is not clear whether the balance between pro-phagocytosis and anti-phagocytosis signals caused by radiotherapy can engulf or not tumor cells under different conditions, resulting in tumor cells sensitivity to radiotherapy. Therefore, it is needed to confirm the balance between these two adverse phagocytosis signals. Thirdly, phagocytosis checkpoint-associated immunotherapy has annoying hematological toxicities, such as anemia, thrombocytopenia, and other immune-associated side effects resulting in limitation of drug dose. Therefore, it is an advisable choice to combine phagocytosis checkpoints-associated immunotherapy with other treatments to reduce these side effects, such as radiotherapy. Fourthly, although there have been some phagocytosis checkpoints associated immunotherapy in clinical patients, there are only two ongoing clinical trials related to combining treatment of radiotherapy and phagocytosis checkpoint-associated immunotherapy one with TTI-621(anti-CD47) (NCT02890368) and the other one with autologous SIRPα-low macrophages (SIRPant-M) (NCT05967416). In the future, researchers maybe should carry out more clinical trials to explore the synergistic effects of radiotherapy and phagocytosis checkpoint-associated immunotherapy.

In conclusion, this review summarizes the influence of radiotherapy on phagocytosis checkpoints in the tumor microenvironment and suggests the optimal modes of combination treatment of radiotherapy and phagocytosis checkpoint-associated immunotherapy by considering diverse therapy regimens to improve antitumor efficacy and tumor patients’ outcomes.
